# Production of l-alanyl-l-glutamine by immobilized *Pichia pastoris* GS115 expressing α-amino acid ester acyltransferase

**DOI:** 10.1186/s12934-019-1077-1

**Published:** 2019-02-02

**Authors:** Yi-Min Li, Jiao-Qi Gao, Xu-Ze Pei, Cong Du, Chao Fan, Wen-Jie Yuan, Feng-Wu Bai

**Affiliations:** 1 School of Life Science and Biotechnology, Dalian University of Technology, Dalian, 116024 China; 2 Division of Biotechnology, Dalian Institute of Chemical Physics, Dalian, 116023 China; 3 Research and Development Center, Dalian Innobio Corporation Limited, Dalian, 116600 China; 4 School of Life Science and Biotechnology, Shanghai Jiaotong University, Shanghai, 200240 China

**Keywords:** l-Alanyl-l-glutamine, α-Amino acid ester acyltransferase, Immobilization, Codon optimization, Recycle

## Abstract

**Background:**

l-Alanyl-l-glutamine (Ala-Gln) represents the great application potential in clinic due to the unique physicochemical properties. A new approach was developed to synthesize Ala-Gln by recombinant *Escherichia coli* OPA, which could overcome the disadvantages of traditional chemical synthesis. Although satisfactory results had been obtained with recombinant *E. coli* OPA, endotoxin and the use of multiple antibiotics along with toxic inducer brought the potential biosafety hazard for the clinical application of Ala-Gln.

**Results:**

In this study, the safer host *Pichia pastoris* was applied as an alternative to *E. coli*. A recombinant *P. pastoris* (named GPA) with the original gene of α-amino acid ester acyltransferase (SsAet) from *Sphingobacterium siyangensis* SY1, was constructed to produce Ala-Gln. To improve the expression efficiency of SsAet in *P. pastoris*, codon optimization was conducted to obtain the strain GPAp. Here, we report that Ala-Gln production by GPAp was approximately 2.5-fold more than that of GPA. The optimal induction conditions (cultivated for 3 days at 26 °C with a daily 1.5% of methanol supplement), the optimum reaction conditions (28 °C and pH 8.5), and the suitable substrate conditions (AlaOMe/Gln = 1.5/1) were also achieved for GPAp. Although most of the metal ions had no effects, the catalytic activity of GPAp showed a slight decrease in the presence of Fe^3+^ and an obvious increase when cysteine or PMSF were added. Under the optimum conditions, the Ala-Gln generation by GPAp realized the maximum molar yield of 63.5% and the catalytic activity of GPAp by agar embedding maintained extremely stable after 10 cycles.

**Conclusions:**

Characterized by economy, efficiency and practicability, production of Ala-Gln by recycling immobilized GPAp (whole-cell biocatalyst) is represents a green and promising way in industrial.

**Electronic supplementary material:**

The online version of this article (10.1186/s12934-019-1077-1) contains supplementary material, which is available to authorized users.

## Background

As the major carrier of nitrogen in the body, l-glutamine (Gln), the most abundant free-form amino acids in the plasma [1], is a conditionally essential amino acid [2, 3]. That is, Gln needs outside supplement for body’s requirements under specific conditions such as strenuous exercise, injury, and infection [4]. However, the widespread clinical applications of free Gln are quite limited due to its unfavorable physicochemical properties [5–7].

An effective approach to overcome intrinsic limitations of Gln is glutamine-containing dipeptides by conjugation with other amino acids [8]. l-Alanyl-l-glutamine (Ala-Gln) and glycyl-l-glutamine (Gly-Gln) are supposed to be two main types of glutamine-containing dipeptides in clinical practice [9]. Ala-Gln has been clinically selected as the most suitable alternative of Gln because of its high thermostability under heat sterilization, highly solubility (568 g/L), and higher decomposition rate [10, 11]. Beyond that, Ala-Gln will be rapidly removed from plasma after parenteral administration without accumulation in tissues and undetectable in urine [12]. These properties indicate that Ala-Gln as an appropriate parenteral nutrition in clinic will become an essential for patients after operation and postoperative recovery [13–15], just like glucose solution or normal saline.

Generally, chemical methods have been utilized for industrial production of Ala-Gln. However, these chemical synthesis pathways have obvious disadvantages [16, 17]. These drawbacks may increase production costs and violate requirements of green chemistry in the 21st century. Compared with traditional chemical synthesis, microbial enzymatic synthesis has the broadest prospect due to the individual advantages such as simple operation, inexpensive raw materials, high conversion efficiency, environmentally friendly process, and recyclability in further industrial-scale applications [18]. Recently, a novel enzyme named α-amino acid ester acyltransferase (SsAet) was found in *Sphingobacterium siyangensis* AJ2458 by Abe et al. [19], which can generate Ala-Gln directly using l-alanine methyl ester hydrochloride (AlaOMe) and Gln. Heterologous expression system of SsAet was constructed in *Escherichia coli* strain, and a total Ala-Gln yield/the molar yield were 69.7 g/L and 67% within 40 min, respectively [8]. In our previous research, OPA, the optimum engineered *E. coli* strain expressing SsAet from *S. siyangensis* SY1 (named OPA), achieved the highest Ala-Gln production with the maximum molar yield of 94.7% and productivity of 1.89 g/L/min. Furthermore, OPA could maintain fast reaction rate (~ 10 min), high Ala-Gln yields and enzyme stabilities after several cell recycling [20].

It is worth noting that the biosafety of products is vital and cannot be ignored in the food, healthcare, and medicine. Although satisfactory results had been obtained with recombinant *E. coli* OPA, endotoxin and the use of multiple antibiotics along with toxic inducer (IPTG) brought the potential biosafety hazard for the clinical application of Ala-Gln. Here, we used the safer host *P. pastoris* to produce Ala-Gln which was added to the qualified presumption of safety (QPS) list by the European Food Safety Authority (EFSA) in 2008 [21]. The safety is further increased by integrated expression of the heterologous gene without antibiotics addition, and the inducer methanol was easily removed by evaporation in the purified process of Ala-Gln. To improve the expression efficiency of SsAet derived from bacteria in *P. pastoris*, codon optimization was first performed. Subsequently, the optimization of the cultivation and reaction conditions were investigated to achieve higher catalytic activity. On this basis, immobilization by agar embedding and recycle were carried out to further improve the catalytic stability and production efficiency. Therefore, a new yeast system using *P. pastoris* GS115 overexpressing SsAet was employed to better the prokaryotic system, which may lay a good foundation for the further industrial-scale clean production of Ala-Gln.

## Materials and methods

### Strains and media

Strains and plasmids used in this study were described in Table 1. For routine use, cultures were maintained on Luria–Bertani (LB) medium (0.5% yeast extract, 1% tryptone, and 1% sodium chloride) agar slants supplemented with the corresponding antibiotics as needed or yeast extract peptone dextrose (YPD) medium (1% yeast extract, 2% peptone, and 2% dextrose) agar slants at 4 °C. For long-term storage, cultures were frozen at − 80 °C in 20% glycerol. The preparation of several different media was required to select and culture the transformants of strain *P. pastoris* GS115 as follows. Regeneration Dextrose Base (RDB) medium (1 M sorbitol, 2% dextrose, 1.34% YNB, 4*10^−5^ % biotin, l-lysine, l-glutamic acid, l-leucine, l-methionine, l-isoleucine each at 0.005%, and 2% agar) for screening His^+^ transformants, minimal dextrose (MD)/minimal methanol (MM) medium (1.34%YNB, 4*10^−5^ % biotin, 2% agar, and 2% dextrose or 0.5% methanol) for selecting the phenotype of transformants with Mut^+^ or Mut^s^ methanol utilization, and buffered glycerol-complex (BMGY)/buffered methanol-complex (BMMY) medium (1.34%YNB, 1% yeast extract, 2% peptone, 4*10^−5^ % biotin, 0.1 M potassium phosphate with pH 6.0, and 1% glycerol or 0.5% methanol) for growth/induction of recombinant strains were prepared on the basis of a manual of *Pichia* Expression Kit [22–24].

**Table 1:** Descriptions of strains and plasmids used in this study

Strain or plasmid	Description	Source
Original strains		
*S. siyangensis* SY1	Template of alpha-amino acid ester acyl transferase gene (*SsAET*)	CGMCC (1.6855)
*E. coli* DH5α	Genetic manipulation	Solarbio (C1100)
*E. coli* TOP10	Genetic manipulation	Solarbio (C1210)
*P. pastoris* GS115	Methylotrophic yeast, capable of metabolizing methanol as individual sole carbon source; a mutation in the histidinol dehydrogenase gene (*HIS4*); histidine auxotroph	Invitrogen (C18100)
Recombinant strains		
TOP10-pUC57-*SsAETp*	pUC57-*SsAETp* plasmid in TOP10; ampicillin-resistant	This study
DH5α-pPIC9-*SsAETp*	pPIC9-*SsAETp* plasmid in DH5α; ampicillin-resistant	This study
DH5α-pPIC9-*SsAET*	pPIC9-*SsAET* plasmid in DH5α; ampicillin-resistant	This study
GS115-pPIC9-*SsAETp* (GPAp)	pPIC9-*SsAETp* plasmid in *P. pastoris* GS115; His^+^ Mut^S^	This study
GS115-pPIC9-*SsAET* (GPA)	pPIC9-*SsAET* plasmid in *P. pastoris* GS115; His^+^ Mut^S^	This study
Plasmids		
pPIC9	Empty vector; *P. pastoris HIS4* vector for methanol-inducible expression of a secreted protein; ampicillin-resistant	Invitrogen (K1710-01)
pUC57-*SsAETp*	pUC57 vector harboring *SsAETp* gene which codon-optimized *SsAET* gene according to codon bias of *P. pastoris*; ampicillin-resistant	This study
pPIC9-*SsAETp*	pPIC9 vector harboring *SsAETp* gene; ampicillin-resistant	This study
pPIC9-*SsAET*	pPIC9 vector harboring *SsAET* gene; ampicillin-resistant	This study

### Construction of expression vectors

*SsAET* gene from *S. siyangensis* SY1 was PCR-amplified using the forward primer *SsAET*-F (*Eco*RI restriction site underlined), and the reverse primer *SsAET*-R (*Not*I restriction site underlined) as shown in Table 2. While both *SsAET* gene and pPIC9 vector were digested with restriction enzymes (*Eco*RI and *Not*I), the expression vector pPIC9-*SsAET* was assembled and stored in *E. coli* DH5α in the presence of ampicillin resistance. Simultaneously, the pUC57-*SsAETp* vector was obtained from Sangon (Shanghai, China), which harbored the codon-optimized gene *SsAETp* with the *P. pastoris* codon usage. Then, *SsAETp* gene was extracted using the same restriction enzymes (*Eco*RI and *Not*I) from the vector pUC57-*SsAETp*. As mentioned above, the expression vector pPIC9-*SsAETp* was generated and stored in *E. coli* DH5α in the presence of ampicillin resistance after being confirmed by enzyme digestion and DNA sequencing.

**Table 2:** Primers used in this work and *Eco*RI and *Not*I sites were lowercase italic

Primers	Sequence (5′–3′)	Purpose
*SsAET*-F	CGACGG*gaattc*ATGAAAAATACAATTTCGTGCC	*SsAET* clone
*SsAET*-R	ATAAGAAT*gcggccgc*CTAATGGTGATGGTGATGATGATCTTTGAGGACAGAAAATTCG	*SsAET* clone
GS115-F	CAGCTTTGATGCCTGAAATC	PCR verification for both
GPA-R	GCATCCTGAAGAAACAATACG	PCR verification for GPA
GPAp-R	CATTCCAGAATTGAACGGAG	PCR verification for GPAp
AOX1-F	CTGGTTCCAATTGACAAGC	DNA sequencing for both
AOX1-R	TGGCATTCTGACATCCTC	DNA sequencing for both

### Transformation and selection for integrated transformants

pPIC9-*SsAET* plasmids, isolated from DH5α-pPIC9-*SsAET*, were linearized with restriction enzyme *Bgl*II and transformed into electro competent cells of *P. pastoris* GS115 following the protocol of Invitrogen. Subsequently, there were three steps to screen for the correct His^+^ Mut^S^ transformants: (1) His^+^ phenotype selection. RDB medium selectively allows the growth of recombinant strains with the integrated *Bgl*II-linearized fragment of pPIC9-*SsAET*, and was utilized to achieve the His^+^ phenotype; (2) Mut^S^ phenotype screening based on His^+^ phenotype. Mut^S^ phenotype rarely metabolizes methanol as a sole carbon source due to the disruption of *AOX1* gene, which was readily selected according to the distinct growth behavior on MD/MM agar plates; (3) His^+^ Mut^S^ transformants were confirmed by PCR analysis using the forward primer GS115-F derived from the AOX1 promoter upstream 120 bp of *P. pastoris* GS115 chromosome 4 (GenBank: FN392322.1) and the reverse primer GPA-R/GPAp-R derived from about 650 bp in the middle of the target gene. After DNA sequencing using AOX1-F and AOX1-R, the His^+^ Mut^S^ transformant was named as GS115-pPIC9-*SsAET* (GPA). Similarly, the GS115-pPIC9-*SsAETp* (GPAp) which includes a codon-optimized *SsAETp* was also constructed as above for further assays. All primers involved are listed from 5′ to 3′ direction in Table 2.

### Expression of target proteins and optimization of induction conditions

*Pichia pastoris* transformants (GPA or GPAp) were cultured using 25 mL BMGY medium in 250-mL flask at 30 °C, 220 rpm for 16–18 h. Then, cells were harvested by centrifuging and resuspended to an initial OD_600_ of 1.0 in 100 mL BMMY fresh medium/500-mL flask with vigorous shaking (250 rpm) at 30 °C. As an inducer, absolute methanol was supplemented to a final concentration of 1.0% (v/v) every 24 h for 120 h (5 days).

To further improve expression levels, the different induction conditions were optimized, including induction temperatures (18 °C, 20 °C, 22 °C, 24 °C, 26 °C, 28 °C, and 30 °C), final concentrations of inducer (0.5%, 1.0%, 1.5%, 2.0%, and 2.5%), and induction time (2 days, 3 days, 4 days, 5 days, and 6 days).

### Ala-Gln production assay

Gln, AlaOMe and Ala-Gln were purchased from Sangon and Aladdin Industrial Corporation (China). Ala-Gln production using GPA or GPAp as the whole-cell biocatalyst was performed as follows: the substrates (100 mM AlaOMe and 100 mM Gln) were directly added to 100 mL cultures after induction, while controlling pH at 8.5 by 6 M NaOH. The enzyme-catalyzed reaction was maintained at 24 °C for 10 min, and then terminated by centrifugation (10,000*g*, 2 min) and heat inactivation (100 °C, 5 min) successively. All samples were temporarily stored at 4 °C for the high performance liquid chromatography (HPLC) analysis.

### Optimization of enzyme-catalyzed reactions

The optimal catalytic reaction conditions were investigated. Reaction temperatures were 20 °C, 22 °C, 24 °C, 26 °C, 28 °C, and 30 °C at pH 8.5; reaction pH was performed among 7.0, 7.5, 8.0, 8.5, 9.0, and 9.5. Besides, various types of amino acid esters were selected as substrates to increase the production of Ala-Gln, such as l-alanine methyl ester hydrochloride (AlaOMe), l-alanine ethyl ester hydrochloride (AlaOEt), l-alanine isopropyl ester hydrochloride (AlaOiPr), l-alanine tert-butyl ester hydrochloride (AlaOtBu), and l-alanine benzyl ester hydrochloride (AlaOBzl). Also, the ratios of AlaOMe/Gln were conducted at 1/1, 1/1.5, 1/2, 1.5/1, and 2/1 to achieve the maximum molar yield in different experimental groups. Subsequently, the different substrate concentrations (AlaOMe-Gln = 150–100, 300–200, 450–300, 600–400, 900–600 mM) with the same ratio of AlaOMe/Gln = 1.5/1 were demonstrated in this study. Besides, in order to further improve the enzyme activity, the reaction was carried out with different additives containing metal ions (K^+^, Na^+^, Ca^2+^, Mg^2+^, Zn^2+^, Fe^2+^, and Fe^3+^), activator (cysteine) and protease inhibitor (EDTA and PMSF) to a final concentration of 10 mM.

### HPLC analysis of Ala-Gln

Activities of SsAet from GPA or GPAp and Ala-Gln formation were detected by HPLC utilizing C18 column (ODS-3, 4.6*250 mm, 5 μm; GL Sciences Inc., Japan). A detailed method was described previously [20].

### Catalytic stability, immobilization, and reusability of GPAp

The catalytic stability of SsAet from GPAp was examined from two aspects. One was stored in the mild pH of 7.2 at 4 °C for 5 days. The other was stored under the optimal reaction pH and temperature (8.5 and 28 °C) for the same time (5 days). Activities of different time points were detected as mentioned above methods.

To promote the possibility of further industrialization, the immobilization of GPAp cells were carried out. The cell pellet was harvested by centrifugation and resuspended in 10 mL phosphate buffer. The prepared cell pellet was instantly mixed with 10 mL 4% (w/v) agar solution which was kept warm in a 55 °C water bath [25, 26]. Then the mixture was slowly dropped in an ice water–oil immiscible solvent to form uniform spherical beads (diameter approximately 3–4 mm). The agar beads were washed and stored with phosphate buffer at 4 °C for the test of the recycle. In this study, 10 cycles of catalytic reactions were performed under the same reaction conditions described above to evaluate the reusability of immobilized cells.

## Results and discussion

### Codon optimization of *SsAET* gene based on *P. pastoris*

Based on our previous research [20], the complete nucleotide sequence of our *SsAET* gene from *S. siyangensis* SY1 was 98% identical with *SsAET* from *S. siyangensis* AJ2458 (GenBank: AB610978.1). Correspondingly, an enzyme SsAet contains 619 amino acids, and the molecular weight was estimated at approximately 71.03 kDa. Compared with the SsAet from AJ2458, three mutated amino acids were found in the unique enzyme SsAet from SY1, which might cause an unanticipated change of enzyme structure and an improved enzyme-catalyzed activity [27, 28].

The target proteins in heterologous hosts are often difficult to function normally because of the codon bias [29]. There are 61 nucleotide triplets (codons) to encode 20 amino acids, but the choice of synonymous codons in different species is strongly biased [30]. A considerable divergence (60%) between prokaryotic and *P. pastoris* codon bias is extremely obvious as shown in Additional file 1: Figure S1. In order to solve the potential problem and improve the expression efficiency, codon optimization was supposed to be a reasonable choice. As an attempt to this end, a 1878 bp DNA sequence (18 bp 6*His tag appended to 1860 bp gene) was optimized for better protein expression in *P. pastoris*. Several rules including but not limited to the following were adjusted: (1) codon usage bias was adjusted to fit the highest expression profile of the target host, and a CAI (codon adaptation index) of 0.8–1.0 is regarded as good for high expression; (2) the ideal percentage range of GC content is between 30 and 70%, perfectly 40–60% and unfavorable GC peaks were removed; (3) undesired motif including restriction enzyme site to be used in sub-cloning and negative cis-acting sites were modified; (4) whole sequence was fine-tuned to increase the translation efficiency and prolong the half-life of mRNA.

**Fig. 1:**
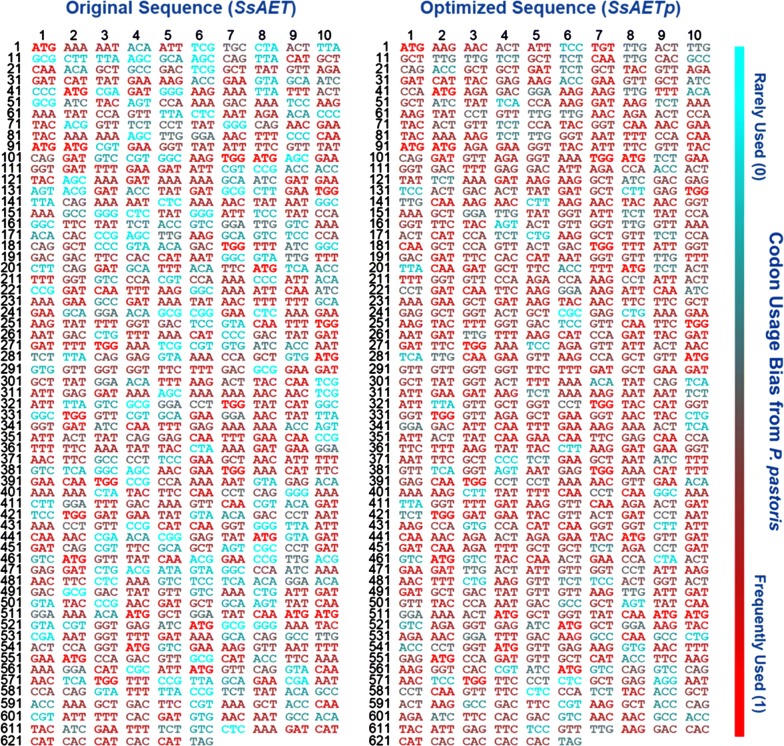
Codon usage bias adjustment of *SsAET* gene. Colors of codons indicated the frequency in host *P. pastoris*. Rarely used codons were shown in cyan and frequently used codons were shown in red. A codon with more red indicated higher frequency in *P. pastoris* for enhanced expression

Therefore, Fig. 1 showed that some infrequent codons in the original gene *SsAET* were selectively replaced by high-frequency codons of *P. pastoris* in the synthesized gene *SsAETp* based on the above principles. As a result, a total of 384 non-preferred codons in the original gene *SsAET* were substituted by the preferred codons of *P. pastoris* in the synthesized gene *SsAETp*, which represents 61.3% of the overall amino acid sequence (Additional file 1: Figure S2). Moreover, CAI was upgrading from 0.69 to 0.89 and the average GC content was adjusted to 40.3%. Thus, the codon-optimized gene was inserted into the pPIC9 vector and transferred into *P. pastoris* to construct the recombinant strain GPAp, while the original gene was used as the control to construct the recombinant strain GPA.

### Screening of the optimized Ala-Gln producer

Several candidates of His^+^ Mut^S^ transformants were found on MD/MM agar plates, which were confirmed by PCR analysis. Lane 2, 18, and 20 all showed the approximately 2000 bp PCR product containing 1200 bp partial vector fragment (120 bp + 650 bp + 1200 bp = 1970 bp), which indicated the *AOX1* gene was accurately replaced by linearized interest gene fragment. After DNA sequencing, His^+^ Mut^S^ transformants were successfully obtained including GPA and GPAp (Additional file 1: Figure S3).

**Fig. 2:**
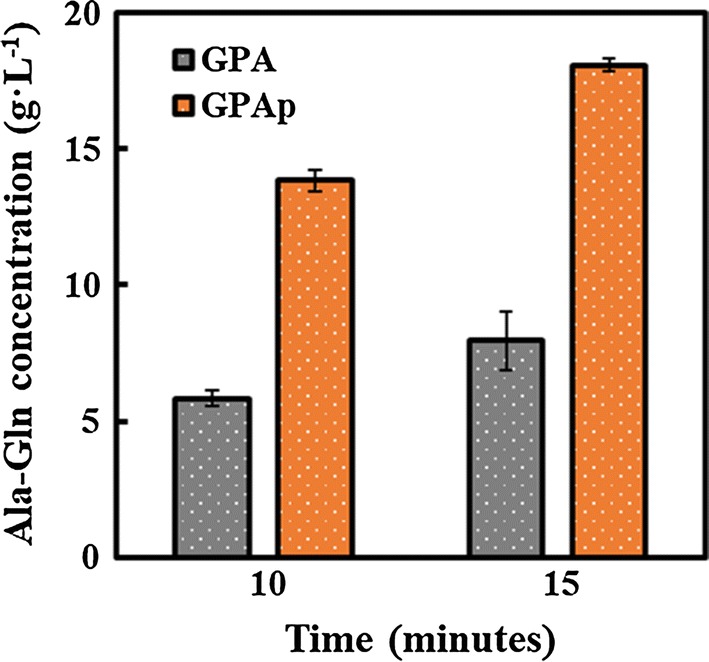
Comparison of catalytic activity between GPA and GPAp to screen for the optimum Ala-Gln producer. GPA and GPAp were denoted in grey and orange, respectively. Samples at two time points were detected by HPLC

After the induction, the supernatant and cells were first used to measure the enzyme activity, respectively. Unexpectedly, the enzyme activity of cells was approximately 10 times than that of the supernatant (Additional file 1: Figure S4). A possible reason is that the size and structure of the protein multimer which forms in the cells block protein secretion. Therefore, GPA and GPAp as a whole-cell biocatalyst were used to synthesize Ala-Gln in subsequent studies. For the sake of screening the optimized Ala-Gln producer, GPA and GPAp were investigated under the same cultural and reaction conditions following “Materials and methods”, and Ala-Gln formation was measured by HPLC. As shown in Fig. 2, the production of Ala-Gln by GPAp was an approximately 2.5-fold increase in comparison with GPA’s when the Ala-Gln accumulation of GPAp reached maximum, indicating the positive effect of codon optimization in heterologous expression. Codon optimization was practically beneficial to improve the expression levels of heterogenous gene, thereby increasing the enzyme-catalyzed activity. As a result, GPAp (whole-cell biocatalyst) was defined as the more suitable Ala-Gln producer for the further study.

### Optimization of induction conditions for Ala-Gln production by GPAp

**Fig. 3:**
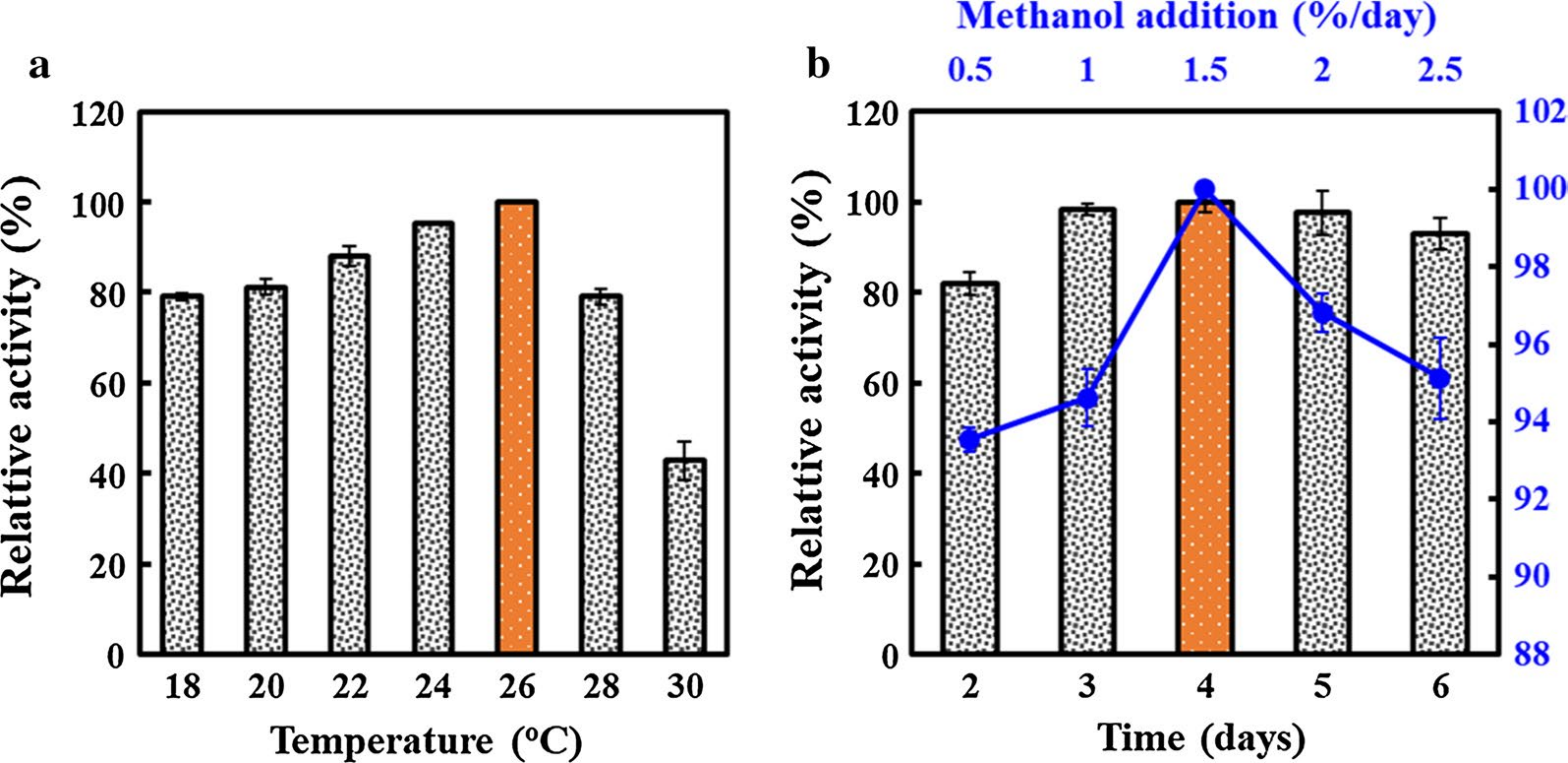
Effects of induction conditions on the catalytic activities of GPAp. **a** Induction temperatures of 18 °C, 20 °C, 22 °C, 24 °C, 26 °C, 28 °C, and 30 °C. **b** Induction time from 2 to 6 days. Blue line showed the different supplemented methanol concentrations of 0.5%, 1.0%, 1.5%, 2.0%, and 2.5%

GPAp, as a direct whole-cell biocatalyst, was used to efficiently synthesize Ala-Gln. In order to further improve SsAet expression levels, a series of parameters in the induced process were worth being explored. Firstly, the effect of induction temperatures was investigated in the range of 18 to 30 °C. The results showed a stable positive correlation between Ala-Gln accumulation (or enzyme activity) and temperature from 18 to 26 °C, and yet an obvious decrease was detected when the temperature was over 26 °C (Fig. 3a), which implied the consistency between the optimal induction temperature and the appropriate cell growth temperature. Secondly, the different induction time was performed within 6 days. Results showed that the highest activity was detected after induction for 4 days, and the enzyme activities were 82.0% and 98.4% of the highest activity for 2 days and 3 days, respectively (Fig. 3b). Additionally, the concentrations of methanol rarely contributed to an enhanced Ala-Gln formation, which means that a moderate supplement was enough to maintain the high activity, as shown in Fig. 3b. Considering both the economy and efficiency, the optimal induction conditions, including temperature (26 °C), duration (for 3 days), and supplemented methanol concentration (1.5%) were determined for GPAp in the future assays. Compared with the prokaryotic induction conditions in the previous study [20], GPAp has two competitive advantages for the Ala-Gln production. On the one hand, it can be induced at room temperature with low equipment requirements; On the other hand, its inducer is easily removed by evaporation to ensure product biosafety.

### Optimization of reaction conditions for Ala-Gln production by GPAp

#### Effects of reaction pH, temperature and substrate

**Fig. 4:**
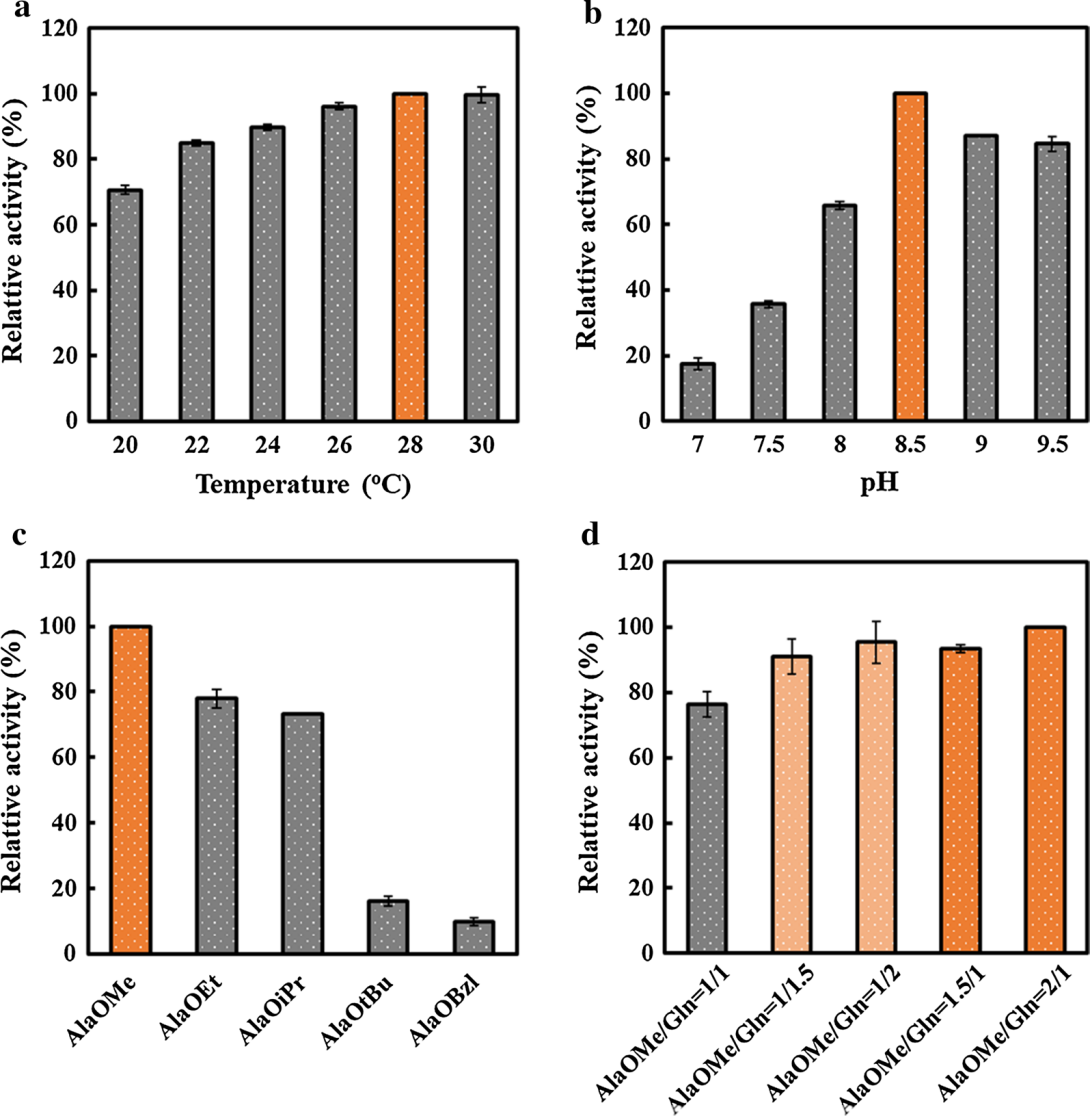
Effects of reaction conditions on the Ala-Gln production. **a** Reaction temperature of 20 °C, 22 °C, 24 °C, 26 °C, 28 °C, and 30 °C at pH 8.5. **b** Reaction pH of 7.0, 7.5, 8.0, 8.5, 9.0, and 9.5 at 24 °C, adjusting with 6 mol/L NaOH. **c** Different amino acid esters, including AlaOMe, AlaOEt, AlaOiPr, AlaOtBu, and AlaOBzl. **d** Different ratios of AlaOMe/Gln of 1/1, 1/1.5, 1/2, 1.5/1, and 2/1

Several reaction conditions were optimized using GPAp under the optimum induction conditions, while catalyzing AlaOMe and Gln to synthesize Ala-Gln. It could be determined that the optimal reaction temperature and pH for Ala-Gln production by GPAp were 28 °C and 8.5, respectively, according to the results of relative activities by HPLC. Additionally, GPAp could steadily maintain over 90% of the highest activity among a wide range of 24 to 30 °C. Similarly, the enzyme activities still remained over 85% of the highest activity when the pH was in the range of 8.5–9.5, whereas the enzyme almost completely inactivated when pH was near or less than 7 (Fig. 4a, b).

Moreover, the effect of different amino acid esters on Ala-Gln generation was detected. Results revealed that the Ala-Gln production using AlaOMe was much higher than others. Unfortunately, the production of Ala-Gln by AlaOBzl was barely detected in our experiments (Fig. 4c). These implied that SsAet from GPAp could selectively bind with preferred amino group bonding sites like methyl ester [31]. As shown in Fig. 4d, we further optimized the ratios of the two substrates (AlaOMe/Gln) to improve the enzyme activity and molar yields. The ratio of AlaOMe/Gln = 2/1 had the highest enzyme activity, which was approximately 1.4-fold higher than that of AlaOMe/Gln = 1/1. The enzyme activity of AlaOMe/Gln = 1.5/1 was in accordance with that of AlaOMe/Gln = 1/2, which still remained over 93% of the highest enzyme activity. The molar yields of Ala-Gln were 47.2%, 57.2%, 59.9%, 58.7%, and 62.8% among different ratios of AlaOMe/Gln = 1/1, 1/1.5, 1/2, 1.5/1, and 2/1. These results demonstrated that the molar yield was significantly improved when either substrate was excess. Hence, taking both the enzyme activity and cost into consideration, the ratio of AlaOMe/Gln = 1.5/1 was determined as the most suitable substrates for the further application.

#### Effects of metal ion, activator and protease inhibitor

Besides, the enzyme SsAet from GPAp was exposed to numerous additives (10 mM) containing various metal ions (K^+^, Na^+^, Ca^2+^, Mg^2+^, Zn^2+^, Fe^2+^, and Fe^3+^), activator (cysteine) and protease inhibitor (EDTA and PMSF) to examine the effect of additives on the catalytic activity. As shown in Table 3, the relative activities of GPAp were not obviously affected by K^+^, Na^+^, Ca^2+^, Mg^2+^, and Zn^2+^, which remained over 99% of the activity compared with blank control. However, the relative activity of GPAp showed a decreasing tendency in the presence of Fe^3+^. In contrast, a positive effect of Fe^2+^, cysteine, EDTA, and PMSF was detected, among which cysteine and PMSF achieved the most increase (approximately 17% and 13%), respectively. These implied that cysteine as a reducing agent might protect the sulfhydryl group of the enzyme from oxidation to improve the GPAp catalytic activity, while PMSF and EDTA could hamper protease and peptidase to avoid the degradation [32].

**Table 3:** Effects of various additives on the activity of GPAp

Additive (10 mM)	Relative enzymatic activity (%)
None	100.00 ± 0.00
K^+^	99.82 ± 0.63
Na^+^	101.52 ± 1.34
Ca^2+^	99.20 ± 1.06
Mg^2+^	101.44 ± 0.72
Zn^2+^	104.63 ± 3.04
Fe^2+^	108.36 ± 1.83
Fe^3+^	91.23 ± 4.39
Cysteine	116.50 ± 1.48
EDTA	107.13 ± 2.81
PMSF	112.59 ± 0.71

#### Accumulation and molar yield under different substrate concentrations

**Fig. 5:**
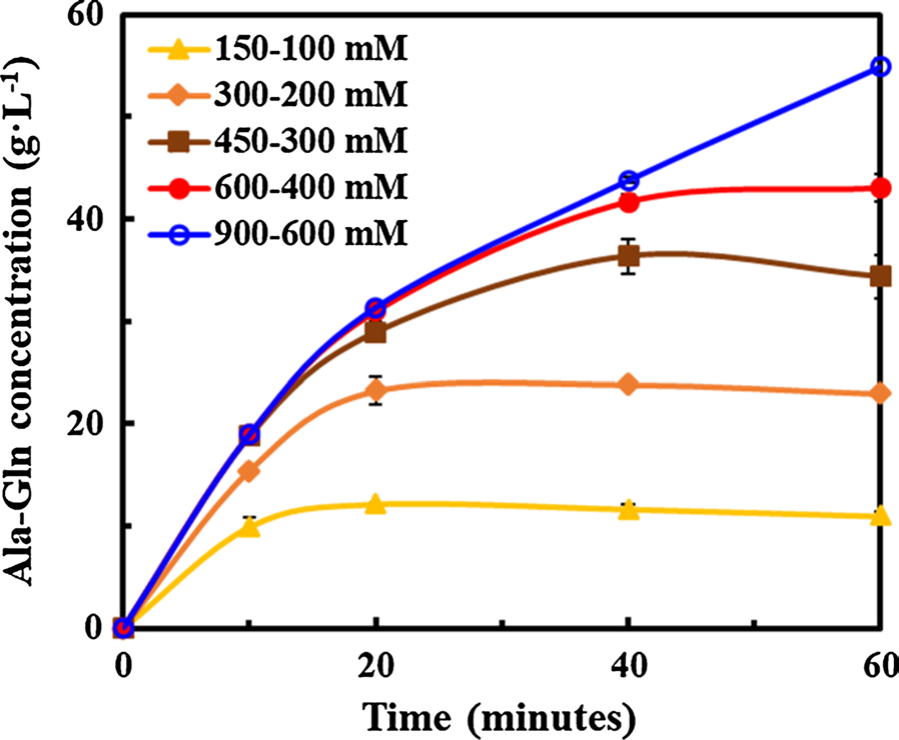
The curves of Ala-Gln concentrations with time in different substrate concentrations

Based on the above optimal reaction conditions, Ala-Gln was synthesized by GPAp using the different substrate concentrations (AlaOMe-Gln = 150–100, 300–200, 450–300, and 600–400 mM) with a ratio of AlaOMe/Gln = 1.5/1. The curve of Ala-Gln production with time is shown in Fig. 5. GPAp could rapidly generate Ala-Gln within 20 min at a lower substrate concentration. As the substrate concentration increased, the reaction time was correspondingly extended to 40 min. And the yields and molar yields under various substrates were 63.5 mM/63.5%, 124.7 mM/62.3%, 181.4 mM/60.5%, and 227.9 mM/57.0%, respectively, which had a slight decrease (about 12–15%) in comparison with previous studies of OPA [20]. Unlike prokaryotic expression system, there is post-translational protein modification in yeast (eukaryotic expression system). Preliminary analysis suggested that the most direct cause of the difference in catalytic activities between OPA and GPAp was glycosylation, including N-linked glycosylation and O-linked glycosylation. Despite not yet being confirmed, four putative N-linked glycosylation sites and five putative O-linked glycosylation sites were predicted referring to the glycoprotein structure of higher eukaryotes [33]. However, only 7 sites with scores over 0.5 were supposed to be the positive glycosylation sites (Additional file 1: Figure S5). In the future experiments, we will adopt measures to avoid protein modification, such as deglycosylation with enzymes or removing the glycosylation sites. And the structures, functions, and modifications of the enzyme (SsAet) from different expressing systems will be specifically studied to illuminate catalytic mechanisms.

Ala-Gln was generally supposed to be an unstable product because of protease or peptidase hydrolysis [34]. Therefore, multiple related coding genes were knocked out in order to reduce the decomposition of Ala-Gln, but cell growth was usually hampered [35]. Fortunately, our results showed that the Ala-Gln generation by GPAp maintained even more stable than that of OPA within 60 min, which could be attributed to the inherent characteristics and growing environments of yeasts (lower pH than neutral pH) with less protease expression or inactivation of many proteases and peptidase [36]. Besides, when further increasing the substrate concentration to 900–600 mM, the GPAp could maintain a consistent production rate of Ala-Gln, which indicated that using the high substrate concentration did not inhibit the catalytic activity of GPAp and increased the feasibility of large-scale industrial production.

### Reusability of immobilized GPAp

#### Stability and immobilization of GPAp

**Fig. 6:**
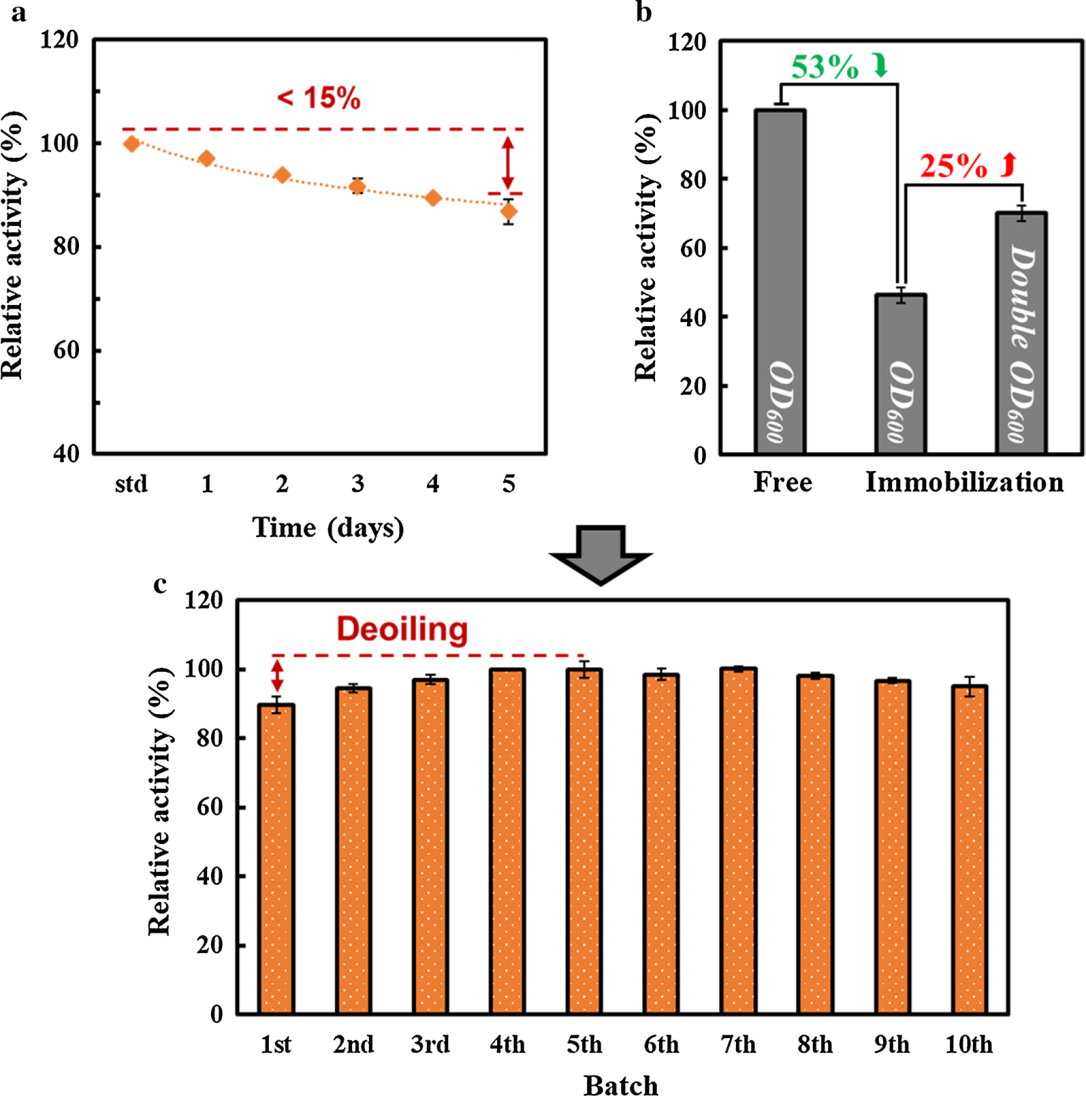
Application of immobilized GPAp by recycling to maintain the stability of Ala-Gln production. **a** The catalytic activities of GPAp were detected daily by HPLC during the 5 days which GPAp was stored at the optimal reaction pH and temperature (8.5 at 28 °C). **b** The catalytic activities were detected and compared among free GPAp with one unit of OD_600_, immobilized GPAp with one unit of OD_600_ and immobilized GPAp with double OD_600_. **c** The reusability of immobilized GPAp was detected at optimum conditions for recycling 10 times

The catalytic stability of GPAp was investigated from two aspects as a prerequisite for subsequent experiments. On the one hand, a decrease of enzyme activity was barely detected when being stored in the mild pH of 7.2 at 4 °C for 5 days. On the other hand, GPAp could remain extremely stable (approximately 90%) under the optimal reaction conditions (pH of 8.5 at 28 °C) for 5 days as shown in Fig. 6a. These results demonstrated that GPAp had the excellent catalytic stability for long-term usage.

Additionally, GPAp was immobilized in agar beads to further improve the catalytic stability and increase the convenience of recycling. The related activities between free GPAp and immobilized GPAp were detected and compared. Results showed that the activity of the immobilized GPAp was half of that of free GPAp using the same biomass (OD_600_). When double biomass (OD_600_) was utilized, the residual activity remained over 70% (Fig. 6b). These results suggested that the higher immobilized temperature (55 °C) which caused the partial GPAp inactivation and mass transfer resistance led to the decreased enzyme activity. One could imagine that continuous increase of immobilized biomass (OD_600_) was practically an effective approach to achieve the similar enzyme activity of free GPAp.

#### Immobilized GPAp by recycling for efficient production

Based on both the catalytic stability and immobilization, the reusability of immobilized GPAp was subsequently investigated to realize a convenient, efficient and effective repeated-cycle batch production of Ala-Gln. Results showed that the catalytic activity of immobilized GPAp could remain terrific stability in the repeated-cycle batch experiments (10 cycles) as shown in Fig. 6c. Moreover, the immobilization was prepared using the method of water in oil (W/O), which retained an oil on the surface of the agar beads. When the oil was gradually removed due to the multiple recycling of beads, a slight increase was found in the first 5 cycles.

*Pichia pastoris* has its unique advantages on Ala-Gln production. As chassis cells, good biosecurity provides a greater possibility for the clinical application. As a catalyst carrier, the enzyme catalytic activity will be better maintained due to mild culture condition and less glycosylation. Consequently, it is anticipated that the industrial production Ala-Gln is likely to be realized with the immobilized GPAp by recycling from the following three aspects: (1) GPAp, as the mainstay of an overall process, is a greener approach without the drawbacks of the chemical method and potential biosafety hazards that caused by endotoxin and toxic inducer. Methanol added in our system is easily removed by evaporation, and an integrated genotype also avoids antibiotics addition; (2) the immobilized GPAp with an enhanced catalytic stability omits the recovery step by centrifuging after each reaction and had the advantages in time-saving, power-saving, and labor-saving; (3) the recyclable immobilized GPAp also avoids a repeated cell cultivation to reduce production costs, which conformed to the sustainable development strategy.

## Conclusion

In this study, SsAet origin from a prokaryotic organism, had been characterised in *P. pastoris* GS115. GPAp with a codon-optimized SsAet was constructed and selected as the whole-cell biocatalyst for the production of Ala-Gln due to its excellent catalytic capacity and reliable biosafety. With the optimization of induction and reaction conditions, the immobilized GPAp for recycling represents a promising approach to achieve economic, efficient and practical Ala-Gln production. Furthermore, we provided a direction and foundation for further studies on enzymatic property of SsAet and indicated a green application for the unique enzyme in the industrial production of Ala-Gln.

## Additional file

**Additional file 1.** Additional figures.

## Abbreviations

Ala-Gln: l-alanyl-l-glutamine
Gly-Gln: glycyl-l-glutamine
Gln: l-glutamine
AlaOMe: l-alanine methyl ester hydrochloride
AlaOEt: l-alanine ethyl ester hydrochloride
AlaOiPr: l-alanine isopropyl ester hydrochloride
AlaOtBu: l-alanine tert-butyl ester hydrochloride
AlaOBzl: l-alanine benzyl ester hydrochloride
SsAet: α-amino acid ester acyltransferase from *Sphingobacterium siyangensis* SY1
*SsAET*: the original gene of SsAet
*SsAETp*: the codon optimized gene of SsAet
OPA: origami 2-pET29a(+)-*SsAET*
GPA: GS115-pPIC9-*SsAET*
GPAp: GS115-pPIC9-*SsAETp*
His^+^: histidine surplus
Mut^S^: methanol utilization slow
Mut^+^: methanol utilization plus
AOX1: alcohol oxidase from *AOX1* gene
YNB: yeast nitrogen base without amino acids
CAI: codon adaptation index
PITC: phenylisothiocyanate
PMSF: phenylmethanesulfonyl fluoride
EDTA: ethylene diamine tetraacetic acid
