# Supplementary material for: Production of l-alanyl-l-glutamine by immobilized Pichia pastoris GS115 expressing α-amino acid ester acyltransferase
Source: Microb Cell Fact. 2019 Feb 2;18:27. doi: 10.1186/s12934-019-1077-1 (PMC6359838; doi:10.1186/s12934-019-1077-1)
Supplement: Supplementary file 1 — Additional file 1. Additional figures. [file 12934_2019_1077_MOESM1_ESM.docx]

**Additional file**

**Production of L-alanyl-L-glutamine by immobilized *Pichia pastoris* GS115 expressing α-amino acid ester acyltransferase**

Yimin Li^1^, Jiaoqi Gao^2^, Xuze Pei^1^, Cong Du^1^, Chao Fan^3^, Wenjie Yuan^1^^[[1]](#footnote-1)^*, Fengwu Bai^4^

^1^School of Life Science and Biotechnology, Dalian University of Technology, Dalian 116024, China

^2^Division of Biotechnology, Dalian Institute of Chemical Physics, Dalian 116023, China

^3^Research and Development Center, Dalian Innobio Corporation Limited, Dalian 116600, China

^4^School of Life Science and Biotechnology, Shanghai Jiaotong University, Shanghai 200240, China


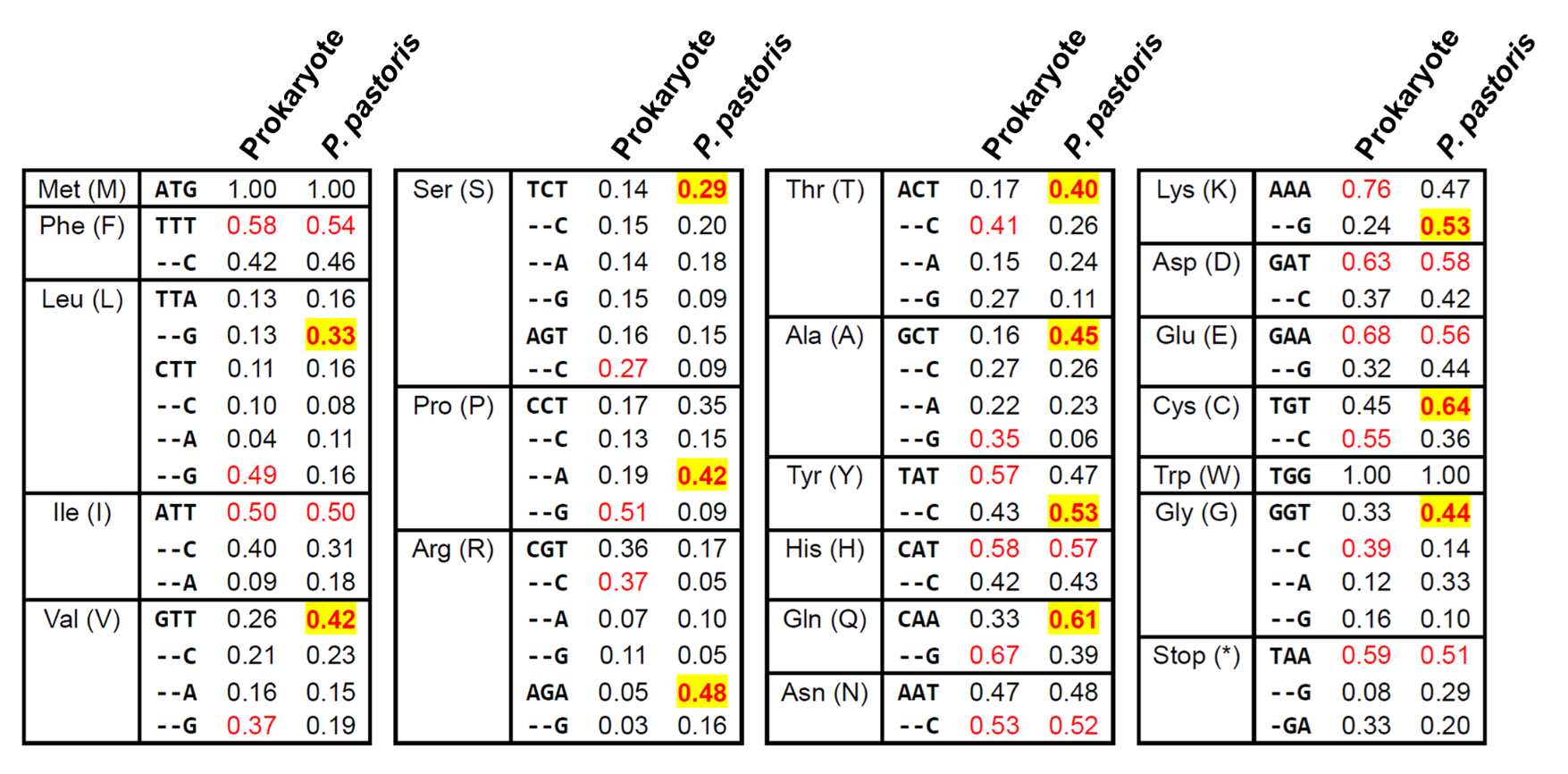


Figure S1. The difference of codon usage bias between prokaryotic and *P. pastoris*. Frequencies of the synonymous codons were demonstrated for each corresponding amino acid. A preferred codon was all represented in red, while highlight indicated that the codon usage bias had changed in *P. pastoris*. The hyphen indicated the same as the above base and the asterisk indicated termination codon.


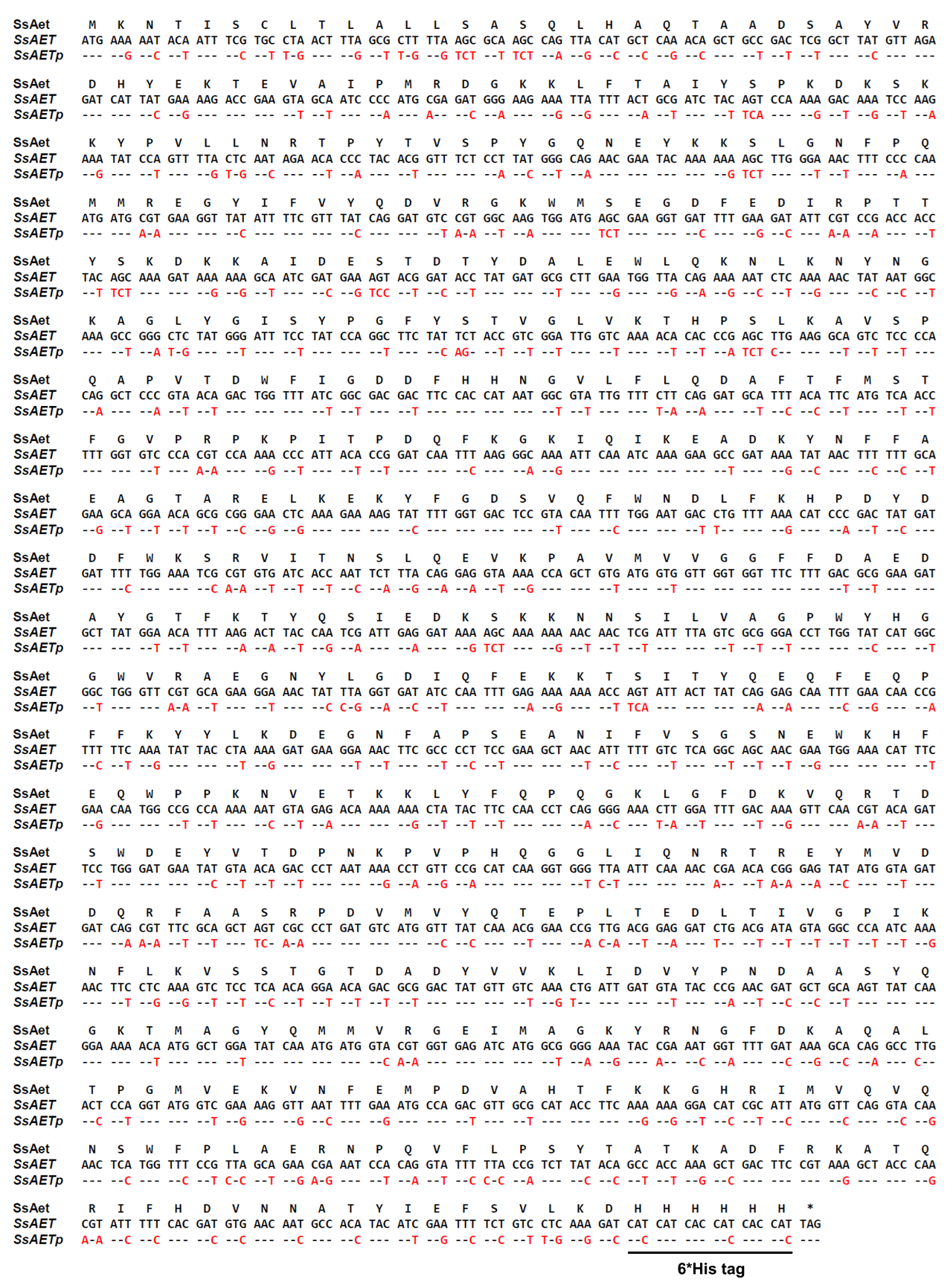


Figure S2. Nucleotide sequences of the original gene *SsAET* and the optimized gene *SsAETp* along with the consistent amino acid sequence. The altered base was shown by the corresponding letter while the consistent base was represented by a hyphen, and the asterisk represented transcriptional stop codon.


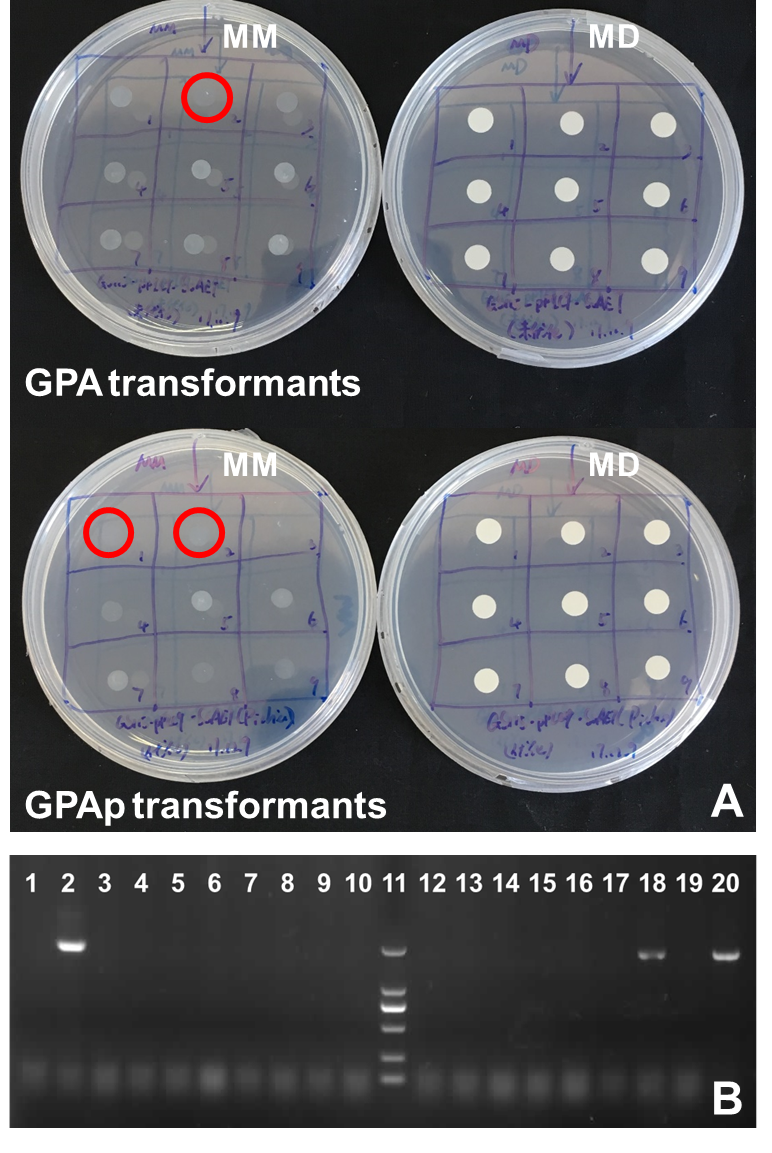


Figure S3. Screening and verification of transformants. (A) His^+^ Mut^S^ transformants were selected according to growth on MD/MM agar plate (partial results). (B) PCR validation of recombinants. Lanes 1-10 were GPA recombinants; Lanes 12-20 were GPAp recombinants; Lane 11 was DL2000 DNA marker.


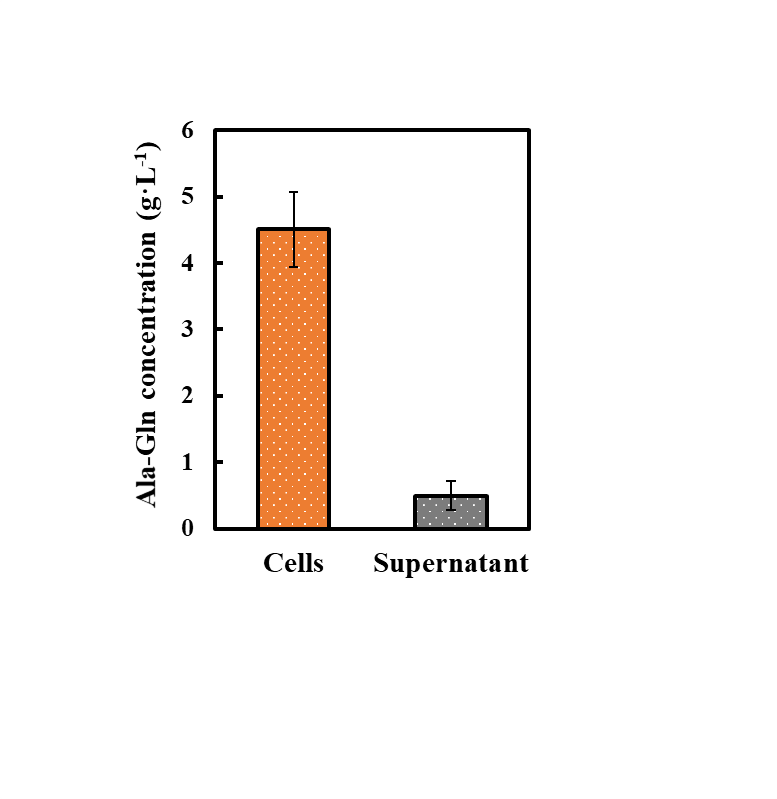


Figure S4. Comparison of catalytic activity between the supernatant and cells. The supernatant and cells were denoted in grey and orange, respectively.


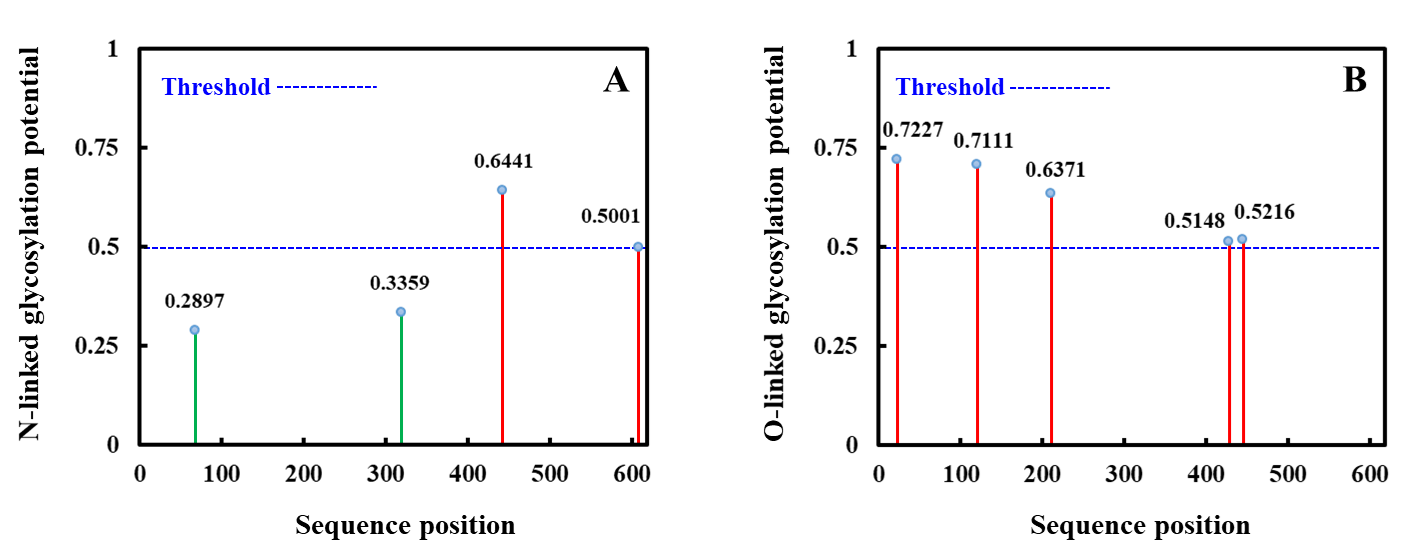


Figure S5. Predicted glycosylation sites across the protein sequence. (A) putative N-linked glycosylation sites. (B) putative O-linked glycosylation sites. A position with a potential (vertical lines) crossing the threshold (horizontal line at 0.5) was predicted as a positive glycosylated site.

1. * Corresponding author. Tel: +86-41184706308; Fax: +86-41184706308. E-mail address: ywj@dlut.edu.cn (wj yuan); [↑](#footnote-ref-1)
